# Alkaptonuria Diagnosis Following a Discectomy: A Case Report

**DOI:** 10.7759/cureus.46644

**Published:** 2023-10-07

**Authors:** Fahad Alhelal, Sami AlEissa, Majed Abaalkhail, Abdullah Alsaeed, Abdullah Alshehri, Fay A Alotaibi, Alanoud Almuhana, Renad M Alzahrani

**Affiliations:** 1 Department of Medicine, National Guard Health Affairs, King Abdulaziz Medical City, Riyadh, SAU; 2 Department of Surgery, Unaizah College of Medicine and Medical Sciences, Qassim University, Riyadh, SAU; 3 College of Medicine, King Saud Bin Abdulaziz University for Health Sciences, Riyadh, SAU

**Keywords:** alkaptonuria, black urine, discectomy, genetic disorder, ochronosis

## Abstract

Alkaptonuria is a rare genetic disorder characterized by the excessive production of homogentisic acid, leading to the formation and deposition of pigment polymers throughout the body. It is extremely rare, affecting only around one in 100,000 individuals. Despite the normal life expectancy, it can cause severe morbidities.

Alkaptonuria is typically managed supportively with pain medication, dietary modifications, and surgical interventions, which are considered to be the gold standard of therapy. Here we present a case of a 33-year-old male with no previous medical or surgical history who presented with severe acute back pain radiating to the left leg. Genetic testing confirmed a homozygous pathogenic variant for alkaptonuria.

This case highlights the challenges in diagnosing alkaptonuria, emphasizing the significance of early detection, and clinical evaluation for improved outcomes. Furthermore, it underscores the need to consider alkaptonuria as a multidimensional disease, necessitating further research to enhance our understanding and develop effective management. Therefore, this study serves as an opportunity for future trials and studies aimed at digging deeper into the intricacies of alkaptonuria to increase our understanding and establish comprehensive management plans for affected individuals.

## Introduction

Black urine disease also known as alkaptonuria is a rare autosomal recessive inborn error of metabolism as defined in 1902 [1]. The defect lies in the catabolism of tyrosine and the deficiency of the homogentisate 1,2-dioxygenase (HGO) enzyme, which is responsible for the formation of homogentisic acid that is, upon contact with air, converted to a pigment polymer that causes the blackish discoloration of the standing urine [2]. This pigment can deposit throughout the body and mainly throughout cartilaginous tissues. This process of deposition/pigmentation takes years and is often presented in adulthood usually beginning from the third decade, in contrast, although usually asymptomatic, some of the earliest signs of presentation are blackish diaper staining and black urine through childhood [3]. Because it is autosomal recessive, chances of development are equally distributed throughout genders. Yet, males tend to have sooner and worse clinical outcomes [4]. Alkaptonuria causes significant morbidity in patients in which patients usually suffer from severe arthritic symptoms along with back pain requiring medical attention [3]. The pigment can also be deposited in valves causing valvular calcification and cardiac manifestations [5]. Using gas chromatography, homogentisic acid can be identified in the urine suggesting alkaptonuria. Disk degeneration plus calcification of imaging can be helpful for diagnosis. Valvular involvement can be assessed by chest x-ray or CT. Histological evaluation will show pigment deposition outside the cells in a sample taken from cartilage. Treatment of alkaptonuria often relies on dietary modification and surgical management. Surgical options include removal of involved disks with fusion and the replacement of the involved joint if necessary [6].

## Case presentation

**Figure 1:**
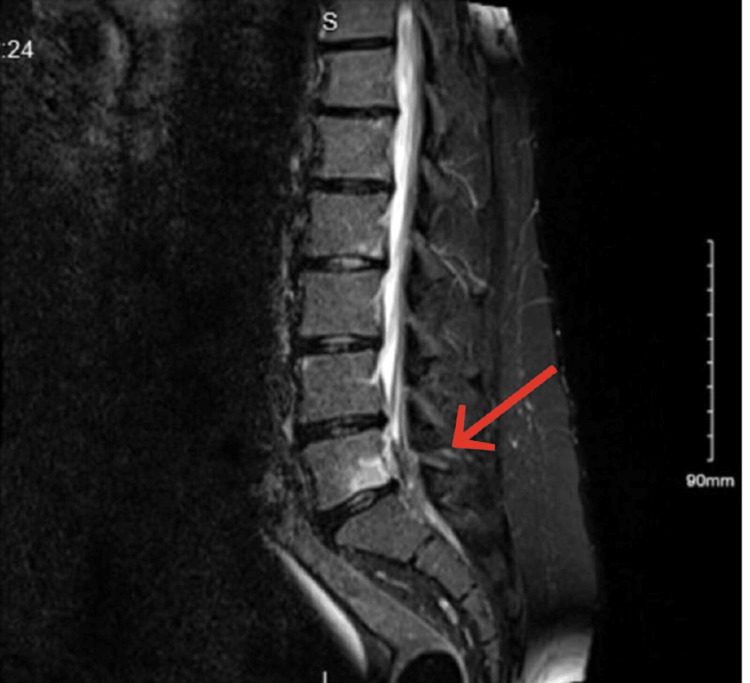
MRI revealed a paracentral left disc herniation at the L5-S1 level, along with Modic type 1 changes on the inferior endplate of the L5 body.

A 33-year-old male with no prior medical or surgical history presented with severe acute back pain radiating to his left leg. The patient's symptoms began two days after lifting a heavy object and getting worse day by day. In addition to weakness and numbness in the left leg initial assessment indicated signs of cauda equina syndrome, leading to an urgent lumbar MRI. The MRI revealed a paracentral left disc herniation at the L5-S1 level, along with Modic type 1 changes on the inferior endplate of the L5 body (Figure 1). 

**Figure 2:**
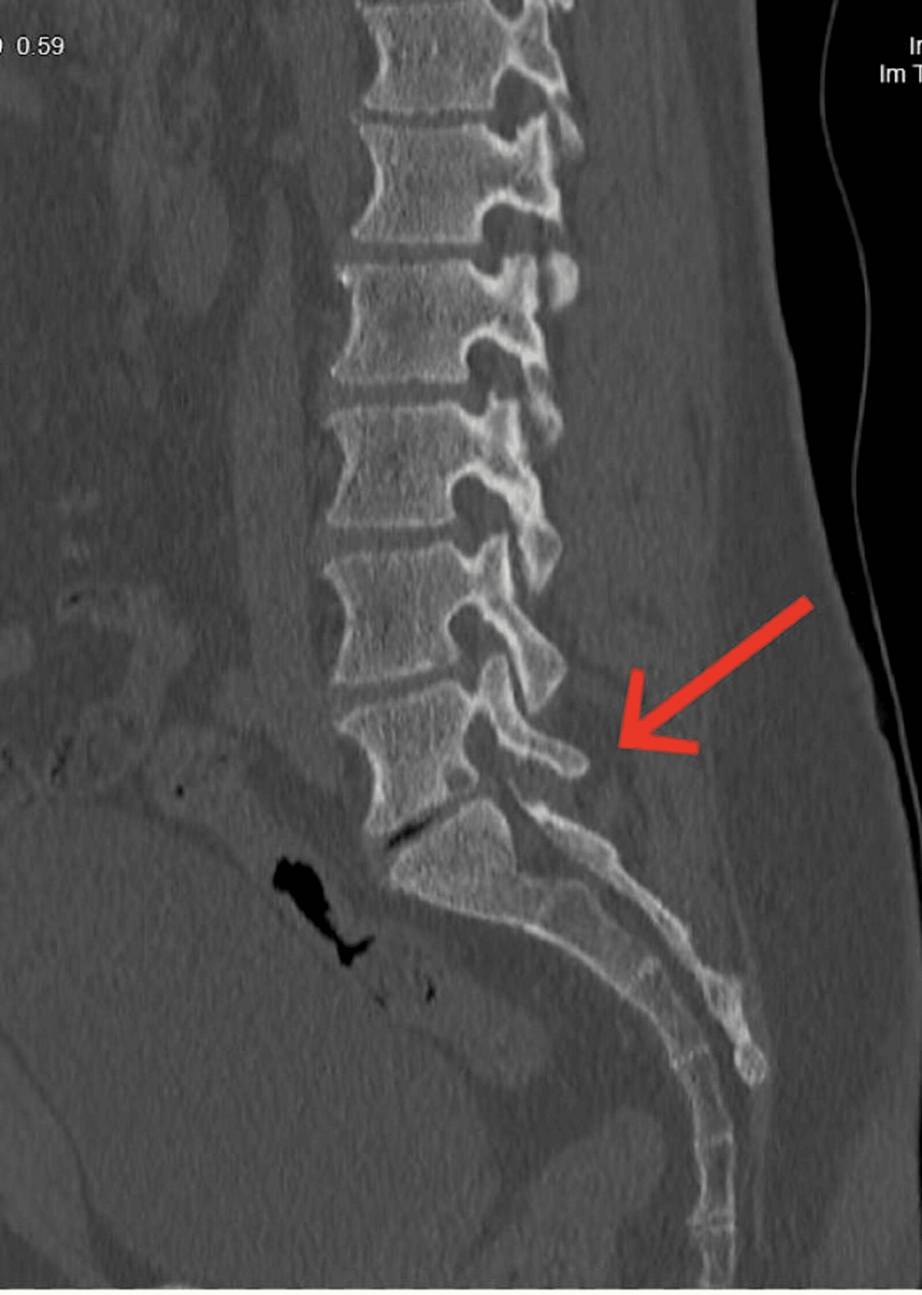
CT scan confirmed the absence of bony osteophytes.

**Figure 3:**
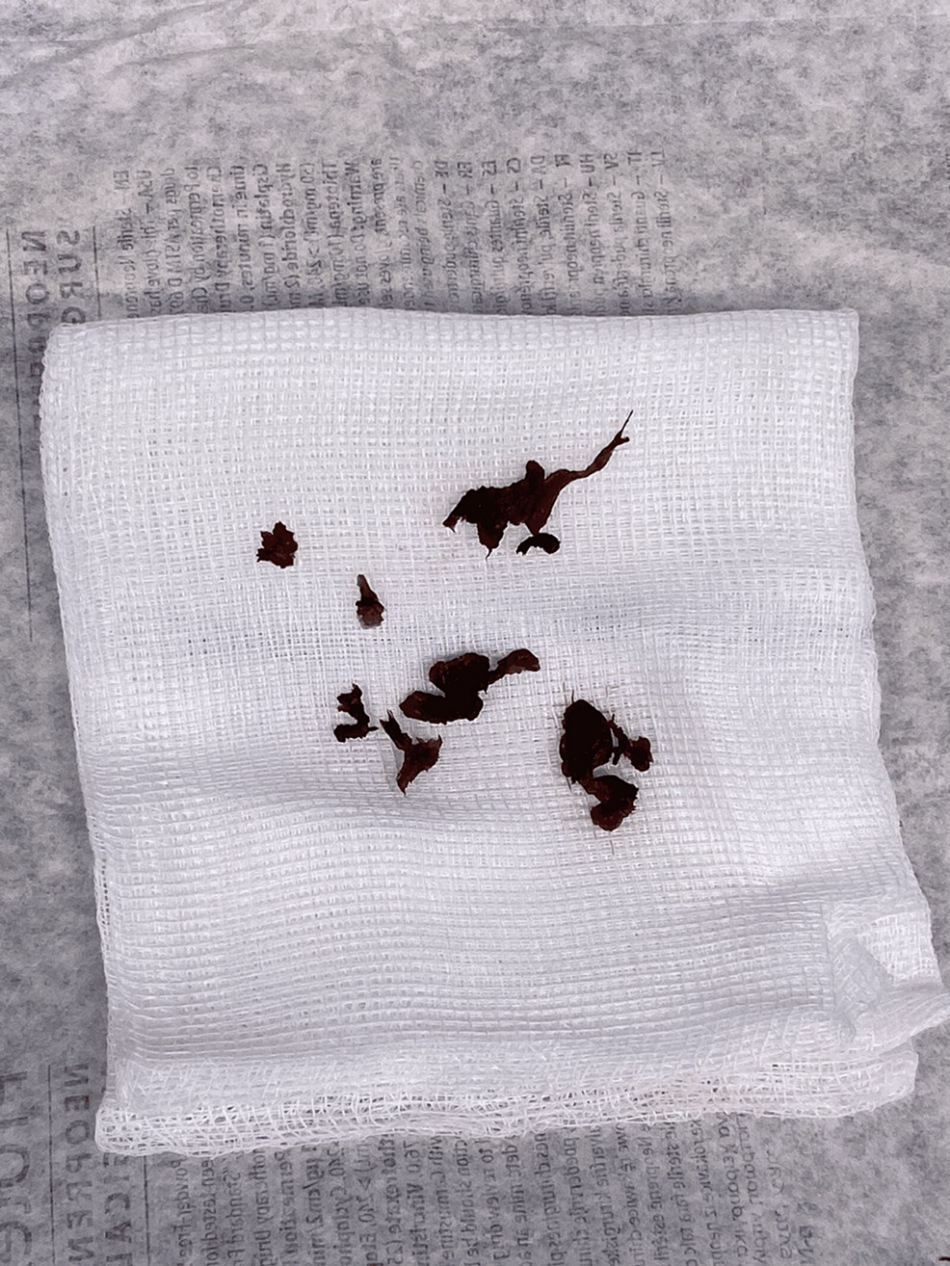
Intraoperative picture of the extracted disc revealed fragile and black tissues.

Further evaluation with a CT scan was performed to assess the presence of any bony abnormalities causing compression and confirmed the absence of bony osteophytes (Figure 2). The patient was initiated on intravenous analgesia and oral pain medications. Upon reassessment, weakness affecting the L5-S1 level was noted, with a strength grade of 3/5, along with decreased sensation and radiating pain throughout the entire leg. Consequently, a decision was made to proceed with a microscopic discectomy of the left L5-S1 disc the following day. The surgical technique was an open approach that employed a minimally invasive posterior technique, and no abnormalities were observed in the skin, connective tissues, or bone. During the surgery, upon incision of the annulus fibrosus, the disc material appeared black and dark in color. After removing all loose disc material, inspection of the extracted disc revealed fragile and black tissues (Figure 3).

Following the surgery, the patient experienced a favorable outcome, with a return of strength and significant improvement in pain and numbness. A retrospective evaluation of the patient's history and physical examination revealed a positive report of dark urine to varying degrees, although no pigmentation was observed in the cartilage or sclera. The patient also mentioned having a sibling in their 30s who presented with similar complaints of back pain and disc disease. Genetic testing was performed on the patient's siblings. The genetic test was positive for a homozygous pathogenic variant which was identified in HGD gene that confirmed the diagnosis of autosomal recessive alkaptonuria in this patient and his siblings. In addition to genetic testing, histopathological testing was conducted. This type of testing involves studying the microscopic structure of tissues obtained from the patient. The purpose of histopathological examination was to confirm the presence of disease-related cellular or tissue changes. The histopathological testing yielded the presence of hyperkeratosis, along with hypergranulosis, and evidence of fibroelastic degeneration of collagen, suggesting disease-specific abnormalities in the affected tissues. This prompted more investigation for alkaptonuria. Therefore, urine homogentisic acid was collected and sent for gas chromatography, with fresh urine appearing normal in color. An echocardiogram (ECHO) was done to rule out any valve abnormalities that usually come with alkaptonuria patients and it was negative. The patient's recovery progressed well, without any complications. After one year of follow-up, it was observed that the patient exhibited no symptoms or signs of the previously diagnosed disease. This outcome indicated a complete resolution or remission of the condition. The follow-up period not only provided reassurance about the patient's well-being but also allowed for a comprehensive assessment of their long-term health status. Overall, the patient's positive clinical outcome in the follow-up, combined with the genetic and histopathological findings, provided valuable information for understanding the nature and potential hereditary aspects of the disease. These insights can contribute to improved diagnosis, treatment, and genetic counseling for the patient and their family members.

## Discussion

Alkaptonuria is an autosomal recessive genetic disease, which was first reported by Garrod as an inborn error of protein metabolism in 1902 [1]. The defective gene has been mapped to chromosome 3 by [1]. Alkaptonuria results in the accumulation of homogentisic acid in connective tissues due to the deficiency of HGD enzyme [1,7]. The accumulated HGA oxidizes to form melanin-like polymer which is responsible for the dark yellow pigmentation of cartilage and other tissues [1]. The majority of the patients are usually asymptomatic until the third and fourth decades [8]. One of the earliest signs is the darkening of the urine upon standing [8]. At the age of 40 years, external signs of bluish-black discoloration, ochronosis, begin to appear [9]. The pigmentation tends to be more prominent in the sun-exposed areas, cartilage, and high sweat gland density sites on the body [8]. Approximately 50% of patients present with ochronotic arthropathy, musculoskeletal manifestation of alkaptonuria, involving weight-bearing joints leading to arthritis and significant back pain [10,11]. Patients often present with back stiffness, with eventual loss of lordosis and exaggerated thoracic kyphosis occurring earlier than the peripheral joints and the spine [12]. Intervertebral calcification at numerous levels and vacuum phenomena with radiolucent gas collections are typical radiologic findings that indicate areas of severe degeneration [12]. Alkaptonuria affects males and females equally. However, males typically experience more severe and earlier-onset arthritic symptoms than females [13]. The diagnosis of alkaptonuria (AKU) is frequently not made until it is identified intraoperatively during orthopedic surgery, when the affected joint shows a distinctive bluish-black coloring, due to the disease's rarity and symptoms that mimic other types of arthritis. Currently, there’s no effective treatment for alkaptonuria. However, low tyrosine and phenylalanine diet, physical therapy, non-steroidal anti-inflammatory drugs (NSAIDs), and antioxidants have been recommended [1,10].

## Conclusions

In conclusion, alkaptonuria is a frequently underdiagnosed disease that requires greater understanding and earlier clinical evaluation. Detecting this condition early is crucial for preventing and treating various systems in the body, as it not only presents with skin changes but also affects the renal, cardiovascular, and liver systems. Therefore, it is essential to raise awareness among healthcare practitioners about the importance of assessing alkaptonuria in cases of unexplained musculoskeletal manifestations, as alkaptonuria is commonly involved in such presentations. Since there is currently no effective treatment for alkaptonuria, it is advised to approach it as a multidimensional disease and further research is necessary. This study provides an opportunity for trials and series to delve deeper and gain more knowledge about this disease.
